# LiDAR-Based 3D Temporal Object Detection via Motion-Aware LiDAR Feature Fusion

**DOI:** 10.3390/s24144667

**Published:** 2024-07-18

**Authors:** Gyuhee Park, Junho Koh, Jisong Kim, Jun Moon, Jun Won Choi

**Affiliations:** 1 Department of Electrical Engineering, Hanyang University, Seoul 04763, Republic of Korea; ghpark@spa.hanyang.ac.kr (G.P.); jhkoh@spa.hanyang.ac.kr (J.K.); jskim@spa.hanyang.ac.kr (J.K.); junmoon@hanyang.ac.kr (J.M.); 2 Department of Electrical and Computer Engineering, College of Liberal Studies, Seoul National University, Seoul 08826, Republic of Korea

**Keywords:** 3D object detection, LiDAR, temporal, motion-aware aggregation, autonomous driving

## Abstract

Recently, the growing demand for autonomous driving in the industry has led to a lot of interest in 3D object detection, resulting in many excellent 3D object detection algorithms. However, most 3D object detectors focus only on a single set of LiDAR points, ignoring their potential ability to improve performance by leveraging the information provided by the consecutive set of LIDAR points. In this paper, we propose a novel 3D object detection method called temporal motion-aware 3D object detection (TM3DOD), which utilizes temporal LiDAR data. In the proposed TM3DOD method, we aggregate LiDAR voxels over time and the current BEV features by generating motion features using consecutive BEV feature maps. First, we present the temporal voxel encoder (TVE), which generates voxel representations by capturing the temporal relationships among the point sets within a voxel. Next, we design a motion-aware feature aggregation network (MFANet), which aims to enhance the current BEV feature representation by quantifying the temporal variation between two consecutive BEV feature maps. By analyzing the differences and changes in the BEV feature maps over time, MFANet captures motion information and integrates it into the current feature representation, enabling more robust and accurate detection of 3D objects. Experimental evaluations on the nuScenes benchmark dataset demonstrate that the proposed TM3DOD method achieved significant improvements in 3D detection performance compared with the baseline methods. Additionally, our method achieved comparable performance to state-of-the-art approaches.

## 1. Introduction

The demand for accurate 3D object detection in robotics and autonomous driving has spurred the development of various LiDAR-based methods [1,2,3,4,5,6,7,8,9,10,11]. However, most existing approaches focus on processing individual LiDAR point sets without considering the temporal data. In real-world scenarios, LiDAR sensors generate a continuous stream of point cloud data. This temporal sequence provides valuable spatiotemporal information which can improve detection performance. Neglecting this temporal aspect limits the ability to leverage sequential data and handle challenges such as occlusion, missing points, and irregular sampling. In order to address these limitations, it is essential to develop methods that effectively utilize the temporal structure of LiDAR data. By considering the temporal order and incorporating motion-aware techniques, we can enhance the accuracy and robustness of 3D object detection, advancing the capabilities of robotics and autonomous driving systems.

In order to address these limitations, recent advancements have extended video object detection techniques to the domain of 3D object detection, and the various techniques are shown in [12,13,14,15,16,17]. The authors of [12,14,16,17] proposed harnessing the temporal aspect of LiDAR data. These include recurrent neural networks (RNNs), such as long short-term memory (LSTM), which capture temporal dependencies and learn the evolution of object features over time. Transformer models, originally developed for natural language processing, have also been adapted to model the temporal relationships in LiDAR data effectively. Furthermore, graph neural networks (GNNs) have shown promise in capturing the spatial and temporal dependencies among LiDAR points, enabling accurate detection and object tracking. These methods utilize graph representations to model point cloud data and exploit the interrelations between points within and across frames.

In this paper, we present a new 3D object detection architecture, referred to as TM3DOD. The key focus of TM3DOD is to leverage the temporal aspects of data to enhance the voxel encoder and aggregate the current BEV features by generating motion features. The proposed TM3DOD method consists of two main components, which are detailed below.

First, TM3DOD takes into account the temporal relationships between LiDAR scans within a set of LiDAR points. This set consists of subsets of LiDAR points collected from temporal laser sweeps. TM3DOD recognizes the distinct subsets when encoding the points within each 3D voxel. The underlying concept is that points obtained from temporal laser sweeps may influence voxel encoding in different ways. In order to address this, we introduce a temporal voxel encoder (TVE), which applies varying attention weights to the LiDAR points in each sweep.

Second, we aim to enhance the current BEV feature map by leveraging sequential LiDAR data. We extract individual BEV feature maps from the consecutive LiDAR voxel and calculate the motion feature by measuring the temporal variation between two adjacent BEV feature maps. Subsequently, we add each motion feature with the corresponding BEV feature and concatenate the aggregated features together. This process ensures that the temporal information is effectively incorporated into the BEV feature map, enabling improved 3D object detection performance.

In order to verify the effectiveness of TM3DOD, we conducted experiments on the widely used nuScenes dataset [18]. We applied TM3DOD to two baseline methods: PointPillar [2] and CenterPoint [19]. The results of our experiments demonstrate significant performance improvements. Specifically, TM3DOD achieved a 4.18% increase in mean average precision (mAP) compared with PointPillar in an ablation study and a 3.61% increase compared with CenterPoint in the nuScenes test benchmark. These performance gains highlight the effectiveness of TM3DOD in enhancing 3D object detection accuracy. Additionally, our proposed TM3DOD method achieved comparable performance to state-of-the-art methods.

The key contributions of this paper can be summarized as follows:

- In this study, we propose a novel 3D object detection architecture called TM3DOD which leverages the temporal aspects of LiDAR data to improve detection performance. TM3DOD focuses on enhancing the voxel encoder and aggregating the current BEV features through the generation of motion features.
- TM3DOD improves the voxel encoder by considering the temporal relationships between LiDAR scans. It also enhances the BEV feature maps by incorporating sequential LiDAR data and calculating motion features. These enhancements enable TM3DOD to better utilize the temporal information for more accurate 3D object detection.
- Our experiments on the nuScenes benchmark dataset confirm the effectiveness of TM3DOD, as it achieved substantial enhancements in 3D detection performance compared with the baseline methods. Moreover, our method achieved comparable performance to state-of-the-art approaches.

## 2. Related Works

### 2.1. 3D LiDAR Object Detection Based on a Single Frame

Overall, 3D object detection techniques using a single snapshot of sensor measurements have undergone rapid advancement and have played a crucial role in autonomous driving. The existing 3D object detectors can be grouped into three categories according to the data used, which are camera-based [20,21,22,23,24,25], LiDAR-based [1,2,3,4,5,6,7,8,9,10,11,26,27], and fusion-based methods [28,29,30,31,32,33]. In this work, we focus on LiDAR-based methods since they are suitable for obtaining accurate depth information and are less sensitive to illumination and weather conditions.

Existing works on single-frame LiDAR-based 3D object detection can be roughly categorized into three approaches: voxel-based methods [1,2,3,4,26], point-based methods [5,6,7], and hybrid methods which combine both approaches [8,9,10,11]. Grid-based methods project the irregular point clouds onto regular grid representations, such as voxels [3] or pillars [2], to extract point features using 2D or 3D convolutional neural networks (CNNs). These methods are more computationally efficient compared with point-based approaches. However, they suffer from quantization loss due to the limited resolution of the grid representation. Examples of grid-based methods include SECOND [1], VoxelNet [3], and PartA2 [26]. Grid-based methods are widely used in autonomous driving applications due to their efficiency. Point-based methods, on the other hand, retain the geometric information of the point cloud by directly processing the unordered points using PointNet [34] or PointNet++ [35]. These methods have the potential to achieve better performance than grid-based methods since they do not suffer from the quantization loss caused by grid representations. However, point-based methods require additional computation costs for sampling and grouping points, which can be computationally expensive. PointRCNN [5], 3DSSD [6], and Point-GNN [7] are examples of point-based methods. Hybrid methods aim to combine the advantages of both grid-based and point-based approaches. They attempt to fuse the merits of grid-based feature extraction and point-based geometric information. Although hybrid methods often achieve better performance than individual approaches, they tend to be more time-consuming compared with grid-based methods. Some examples of hybrid methods include FastPointR-CNN [8], STD [9], PV-RCNN [10], and SA-SSD [11].

### 2.2. 3D LiDAR Object Detection Based on Multiple Frames

Despite the rapid advancements in 3D object detection using single LiDAR point sets, these methods have limitations due to the lack of utilization of temporal information in sequence data. In order to overcome the limitations of single point-based 3D object detection, recent methods [12,13,14,15,16,17,27,36] have focused on utilizing sequence data to exploit temporal information. To capture a spatiotemporal representation of point clouds, some approaches [12,13] employ recurrent neural networks (RNNs) such as LSTM [37] and GRU [38]. VelocityNet [15] employs deformable convolution to align temporal features. Other methods [16,17] utilize transformer models known for their superior performance in sequential tasks such as natural language processing. Transformers excel at capturing long-range dependencies and complex temporal relationships. As LiDAR point clouds are affected by egomotion, which can degrade 3D object detection performance, most approaches mitigate this influence by incorporating egomotion transformation using GPS sensors. However, accurately detecting dynamic objects which undergo siginificant amounts of motion remains a challenge despite aligning static objects across frames. In order to address these challenges, 3DVID [13] introduces a temporal transformer attention scheme to align moving objects. TCTR [14] also has a similar architecture, using temporal information based on a transformer.

In our proposed method, TM3DOD, we further enhance the temporal aspect by utilizing temporal motion information in both the voxel and BEV domains. Previous research has suggested feature aggregation in the BEV domain. However, this has a limitation; the BEV feature does not contain all of the data information of LiDAR due to encoding. In order to overcome this, we aim to improve feature representation over time by aggregating LiDAR voxels and current BEV features through motion feature generation. By incorporating these enhancements, TM3DOD aims to enhance the accuracy and robustness of 3D object detection. It effectively leverages temporal information and utilizes motion-aware feature aggregation to improve object detection across frames.

## 3. Proposed Method

In this section, we present details of the structure of the proposed method. We first provide a comprehensive overview of the proposed architecture for 3D object detection, referred to as TM3DOD. Afterward, a temporal voxel encoder (TVE) is presented which considers the temporal correlations between LiDAR data in a voxel domain. Finally, we explain the motion-aware feature aggregation network (MFANet), which combines BEV features by using an attention mechanism.

### 3.1. Overall Architecture

**Figure 1:**
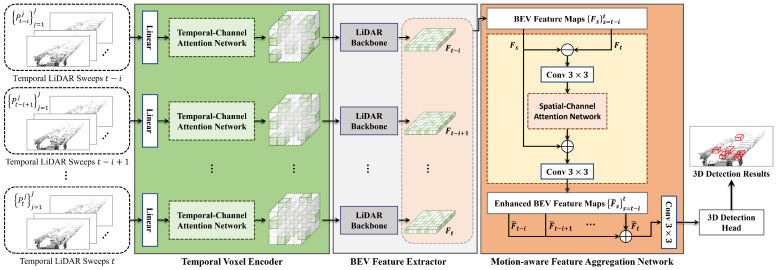
Overall architecture of the proposed TM3DOD method.

The overall architecture of our proposed TM3DOD method is illustrated in Figure 1. TM3DOD comprises two main modules: a temporal voxel encoder (TVE) and motion-aware feature aggregation network (MFANet). These modules are designed to effectively incorporate the temporal aspects of LiDAR data and enhance the performance of 3D object detection.

In the TVE module, the input consists of consecutive LiDAR points obtained from temporal laser sweeps. The goal of the TVE module is to produce voxel representative features over time. In order to achieve this, the TVE module employs a temporal channel attention network, which assigns varying attention weights to the LiDAR points within each voxel based on their temporal relationships. This attention mechanism enables the encoder to focus on the important channels and timestamps of point features while being robust against irrelevant or noisy points within the voxel. By leveraging the temporal relationships between LiDAR scans, the TVE module effectively encodes the points within each voxel, taking into account their varying influences across different laser sweeps.

Once the LiDAR points have been organized into voxels using the TVE module, shared-weight LiDAR backbone networks are utilized to process *N* sequential point sets and generate BEV spatial feature maps. This backbone network plays a crucial role in extracting discriminative features from the BEV representations, which are essential for accurate 3D object detection.

Following utilization of the TVE module and the LiDAR backbone networks, the MFANet module is employed for motion-aware feature aggregation. This module aims to enhance the current feature map by considering both the current feature map and the previously generated feature maps. It involves generating motion features by quantifying the temporal variation between two consecutive visual feature maps. By capturing the changes in feature representations over time, the motion features provide valuable information about object dynamics and improve the discriminative power of the feature maps. In order to incorporate the motion features into the current feature map, each motion feature is added to the corresponding BEV feature, and the resulting features are added. This enables the TM3DOD method to effectively aggregate and integrate the temporal information into the feature maps, leading to enhanced 3D object detection performance.

In summary, the proposed TM3DOD method leverages the TVE module to capture the temporal relationships between LiDAR scans, the LiDAR backbone networks for feature extraction from BEV representations, and the MFANet module for motion-aware feature aggregation. By effectively incorporating the temporal aspects of LiDAR data, TM3DOD aims to improve the accuracy and robustness of 3D object detection in dynamic environments.

Please refer to Figure 1 for a visual representation of the overall TM3DOD architecture.

### 3.2. Temporal Voxel Encoder (TVE)

**Figure 2:**
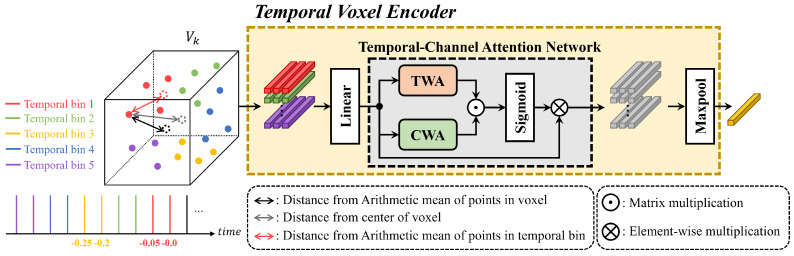
The proposed network of the temporal voxel encoder (TVE).

**Point Feature Extraction:** To encode point features in the temporal domain, we divided the points within the temporal LiDAR sweeps into fixed time intervals. This process is illustrated in Figure 2. For each voxel, let $P_{t-i}^{j}$ represent the ${\left(t-i\right)}^{th}$ point in the $j^{th}$ time interval. To capture the temporal relationships, we calculated the relative position of each point $P_{t-i}^{j}$ with respect to the mean position of the points within the voxel at time *j*, denoted as $\left(x-x_{d},y-y_{d},z-z_{d}\right)$. Here, (x,y,z) represents the position of $P_{t-i}^{j}$, and $\left(x_{d},y_{d},z_{d}\right)$ represents the mean position of the points at time *j*. This distance computation was performed for all points within the voxel and across all voxels. The resulting point features were then processed through a linear layer to extract relevant information.

**Temporal Channel Attention Network (TCANet):** In TCANet, we generated attentive voxel features by focusing on the crucial channels and timestamps of the point features. Let $V^{k}\in R^{N^{k}\times C}$ be a set of point features of the $k^{th}$ voxel and $v_{j}^{k}\in R^{N_{j}^{k}\times C}$ be a set of point features of the $j^{th}$ sweep in the $k^{th}$ voxel (i.e., $N^{k}=\sum_{j=1}^{J}N_{j}^{k}$). Note that *N* is the number of LiDAR points in a voxel. Then, we applied temporal-wise attention (TWA) and channel-wise attention (CWA) to extract the temporal-wise response $U^{k}\in R^{J}$ and channel-wise response $E^{k}\in R^{C}$, respectively. First, in TWA, the temporal response $H^{k}\in R^{J}$ is produced through max pooling across the point-wise dimension and channel-wise dimension on each $v_{j}^{k}$, which is a subset of $V^{k}$. Similarly, the channel response $G^{k}\in R^{C}$ is produced through max pooling across the point-wise dimension of $V^{k}$. In order to explore temporal- and channel-wise correlation, following the attention mechanism called SENet [39], TWA and CWA extract the temporal-wise attention $U^{k}\in R^{J\times 1}$ and $E^{k}\in R^{1\times C}$ as follows:

(1) $$\begin{array}{c} U^{k}=W_{TWA}^{2}\delta \left(W_{TWA}^{1}H^{k}\right) \end{array}$$

(2) $$\begin{array}{c} E^{k}=W_{CWA}^{2}\delta \left(W_{CWA}^{1}G^{k}\right) \end{array}$$

where $W_{\cdot }^{1}$ and $W_{\cdot }^{2}$ are the weight parameters of two fully connected layers in each attention module and δ is the ReLU function. Next, we obtain the attention matrix $M^{k}\in R^{J\times C}$ as follows:

(3) $$\begin{array}{c} M^{k}=\sigma \left(U^{k}⊙E^{k}\right)=\left[m_{1}^{k},\ldots ,m_{J}^{k}\right]\in R^{J\times C} \end{array}$$

where ⊙ denotes the matrix multiplication operation and σ denotes the sigmoid function, which is employed to normalize the values of the attention matrix to the range of [0,1]. The temporal-wise weighted feature $Z^{k}$ for $k^{th}$ voxel is calculated as follows:

(4) $$\begin{array}{c} Z^{k}=maxpool\left[m_{1}^{k}⊗v_{1}^{k},\ldots ,m_{J}^{k}⊗v_{J}^{k}\right]\in R^{C} \end{array}$$

where ⊗ denotes the element-wise multiplication operation and max pooling is performed across the voxel-wise dimension.

### 3.3. Motion-Aware Feature Aggregation Network (MFANet)

**Figure 3:**
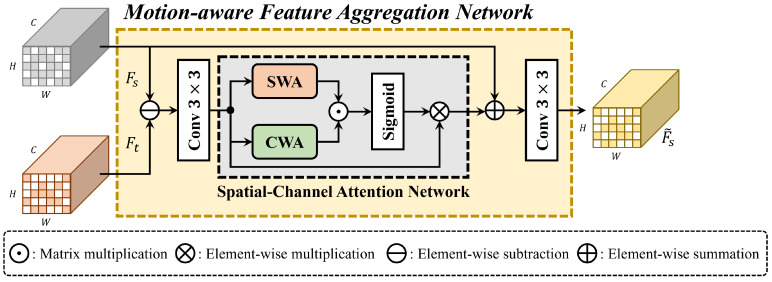
Proposed network of motion-aware feature aggregation network (MFANet).

The goal of MFANet is to leverage the temporal information from consecutive LiDAR sweeps and incorporate it into the current feature map to improve the accuracy of 3D object detection. MFANet is illustrated in Figure 3. The BEV features, denoted as $F_{s}$ from times t−i to *t*, are extracted from the LiDAR backbone network and passed to MFANet. Note that $F_{t}$ indicates the BEV feature at time t, and this is the current BEV feature.

In order to generate each motion feature map in the MFANet, a two-step process is followed. First, the differences between two adjacent BEV feature maps are calculated. These difference maps are then passed through a convolutional network to extract the motion features $M_{t}$:

(5) $$\begin{array}{cc} M_{s} & =({\mathrm{conv}}_{3\times 3}\left(F_{s}-F_{t}\right)), \end{array}$$

In order to further enhance the motion features and enable the network to focus on important spatial and channel-wise information, the spatial-wise attention (SWA) and channel-wise attention (CWA) mechanisms are employed. In SWA, the spatial-wise attention weights are obtained by performing max pooling across the channel-wise dimension of the motion feature maps. This allows the network to highlight important spatial regions in the motion features. Similarly, in CWA, the channel-wise attention weights are obtained by performing max pooling across the pixel-wise dimension of the motion feature maps. This enables the network to emphasize important channels in the motion features. In order to generate the attention weights, we used a sigmoid function which normalized the feature maps between 0 and 1, similar to the TCANet approach. In order to incorporate the generated attention weights into the current feature map, they were multiplied element-wise with the corresponding difference map of two adjacent BEV feature maps:

(6) $$\begin{array}{cc} \tilde{M_{s}} & =\sigma \left(CWA\left(M_{s}\right)⊙SWA\left(M_{s}\right)\right)⊗M_{s}, \end{array}$$

where $\tilde{M_{s}}$ denotes the enhanced motion feature from CWA and SWA. Finally, the enhanced motion feature was then added to the corresponding BEV feature map and passed into the convolution network to generate the final aggregated feature maps $\tilde{F_{s}}$:

(7) $$\begin{array}{cc} \tilde{F_{s}} & ={\mathrm{conv}}_{3\times 3}\left(\tilde{M_{s}}+F_{s}\right), \end{array}$$

## 4. Experiments

### 4.1. nuScenes

The nuScenes dataset is a popular benchmark for 3D object detection in the field of autonomous driving. It comprises a large collection of annotated driving scenes specifically designed for evaluating 3D object detection algorithms. The dataset consists of more than 1000 driving scenes, with 700 scenes designated for training, 150 for validation, and 150 for testing. In the context of 3D object detection, the nuScenes dataset provides annotated point cloud data captured by LiDAR sensors. The LiDAR point clouds are represented by coordinates (x, y, z), a reflectance value (r), and the time lag (Δ t) between each point and the corresponding keyframe. In order to enhance the density of the dataset, the point clouds from the previous 10 sweeps were utilized, resulting in approximately 300,000 point clouds per scene. This allowed for a comprehensive representation of the scene and enabled accurate 3D object detection. The dataset includes annotations for various object categories relevant to autonomous driving, such as bicycles, buses, cars, motorcycles, pedestrians, trailers, trucks, construction vehicles, and traffic cones. For the 3D detection performance metrics, we used the mAP and nuScenes detection score (NDS) [18] as suggested for the nuScenes dataset.

### 4.2. Implementation Details

**Architecture:** We utilized two widely used 3D LiDAR object detectors as single-frame baseline detectors. First, we adopted PointPillar [2] in our framework since it has the advantage of low computation with 2D BEV representation. Second, we chose CenterPoint [19] with a VoxelNet backbone as a baseline so that we could produce a detailed 3D representation. The range of the point cloud was within [−51.2,51.2]×[−51.2,51.2]×[−5.0,3.0] m on the (x,y,z) axis, which was voxelized, with each voxel size being 0.2×0.2×8.0 m in the PointPillar backbone. On the other hand, we considered the points located within [−54.0,54.0]×[−54.0,54.0]×[−5.0,3.0] m on the (x,y,z) axis in the VoxelNet backbone. The grid size for grouping points was set to 0.075×0.075×0.2 m, and this partitioning led to a voxel structure 1440×1440×40 in size. To implement the TVE module, we used the input features which contained original geometric point information and the distance from the mean of the points in the temporal bin. We set bin periods of 2 and 5 to extract motion information at the point level.

**Training and Inference:** PointPillar [2] and CenterPoint [19] were used as single-frame baselines to implement the proposed TM3DOD method. We trained the entire network using a one-cycle learning rate policy for 50 epochs, with a maximum learning rate of 0.003 for PointPillar and 0.001 for CenterPoint, using the whole nuScenes training set. The sequence length was set to 2 s, which contained 4 keyframes. The proposed model was trained using data augmentation methods, including the random flipping, rotation, scaling, and ground truth box sampling used in [1]. We utilized the loss function suggested for the anchor-based [2] or anchor-free baseline [19] to train the proposed model. An Adam optimizer was used to optimize the loss function. We trained our proposed method on 4 NVIDIA RTX 2080 Ti GPUs with a batch size of 8 in the PointPillar-based model and a batch size of 4 in the CenterPoint-based model.

### 4.3. Performance Analysis

In Table 1, we present a comparison between the performance of the proposed TM3DOD method and several state-of-the-art 3D object detectors [2,6,19,33,40,41,42,43,44,45,46,47] on the nuScenes test benchmark.

The results demonstrate that the proposed TM3DOD method achieved superior performance compared with the existing 3D object detection methods. Specifically, when compared with the CenterPoint baseline, TM3DOD exhibited a notable improvement of 2.64% in terms of NDS and 3.61% in terms of mAP. These improvements highlight the effectiveness and robustness of the proposed method in accurately detecting 3D objects in LiDAR-based systems.

Overall, the experimental results presented in Table 1 demonstrate that the proposed TM3DOD method outperformed the existing state-of-the-art approaches and established a new benchmark for 3D object detection in LiDAR-based systems, specifically on the nuScenes dataset.

### 4.4. Ablation Study

In this section, we present some ablation studies to verify the effect of the ideas in the proposed TM3DOD method. Note that our ablation study was performed using PointPillar [2] as a single-frame baseline. Since nuScenes has many training samples compared with KITTI (28,130 versus 3712), the multiple models were trained with time efficiency using the downsampled training set (namely the mini-training set) and validated using the fully valid set. The mini-training set contained about 4000 samples uniformly sampled from the fully trained set.

First, Table 2 demonstrates the effectiveness of the proposed modules used in TM3DOD. We trained the model in combination with the baseline and proposed modules (i.e., TVE and MFANet) while using the mini-training set to validate each of the proposed modules. When MFANet was added to the baseline, it offered 2.19% and 1.32% performance gains over the baseline in the mAP and NDS metrics, respectively. For both TVE and MFANet, it yielded a 4.18% gain in mAP and a 2.40% gain in NDS over the single-frame baseline. Regarding computational complexity aspects, the runtime increased by 13 ms and 8 ms for the MFANet and TVE modules, respectively.

In our analysis, we investigated the impact of the components of the TVE module on the performance of the model. We conducted experiments and summarized the results in Table 3. To ensure a fair comparison, we trained the single-frame baseline model using the mini-training set. We conducted an ablation study on the TVE module using two steps. First, we verified the effectiveness of temporal channel attention. Second, we analyzed the performance according to the temporal bin period in the TVE module. When we added the temporal channel attention mechanism to the voxel encoder, we observed an improvement in the mean average precision (mAP) compared with the baseline model. It improved the mAP by 1.25%. We also show the contribution of the temporal bin period in Table 3. The performance increased by 1.54% when using a temporal bin period of two and 2.67% when using a temporal bin period of five over the baseline. Furthermore, we explored the effect of different temporal bin periods on performance. By setting the temporal bin period to two and five, we observed additional improvements in the mAP by 3.29% over the baseline. These results demonstrate the effectiveness of incorporating the temporal channel attention mechanism and optimizing the temporal bin period in the TVE module, leading to improved performance in 3D object detection tasks.

**Figure 4:**
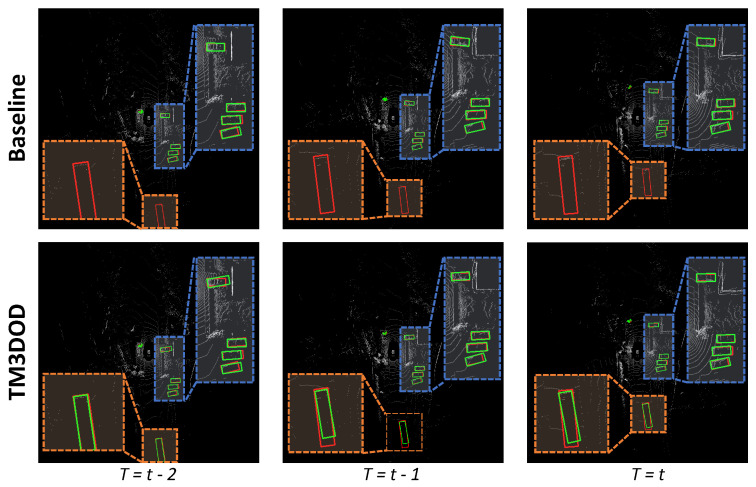
Comparison of qualitative results between the single-frame baseline method and the proposed method on the nuScenes validation dataset.

For further analysis, we compared the qualitative results between the single-frame baseline method and the proposed method on the nuScenes validation dataset. In Figure 4, the red boxes represent the ground truth, and the green boxes represent the predictions. In order to verify the effectiveness of the proposed method, we compared the results for a frame that had both static objects and moving objects. In the baseline method, the static objects (blue boxes) were detected well, while the moving objects (orange boxes) were not detected. In contrast, our proposed method detected both static and moving objects.

## 5. Conclusions and Future Work

In conclusion, the TM3DOD framework presents a novel approach to 3D object detection in LiDAR-based systems. By incorporating the temporal voxel encoder (TVE) and motion-aware feature aggregation network (MFANet) modules, TM3DOD effectively utilized the temporal information from consecutive LiDAR sweeps to improve the accuracy and performance of object detection. The TVE module captured motion over time with its temporal channel attention network, enhancing the encoder’s ability to focus on important points and improve robustness. The MFANet module further improved the detection results by aggregating the motion features using the current feature map. Our experiments on the nuScenes dataset demonstrated the effectiveness of TM3DOD compared with LiDAR-only baselines. The proposed method achieved significant improvements in accuracy and showed comparable performance to state-of-the-art methods. Nevertheless, there were some performance decreases for small or stationary objects such as pedestrians, traffic cones, and barriers. Even if we use the multi-frame LiDAR point cloud data, it is difficult to improve performance for such categories, which show similar characteristics in terms of their LiDAR data aspects. As a future work, we need to research 3D object detection with sensor fusion to overcome these difficult cases.

## Figures and Tables

**Table 1:** Performance comparison on nuScenes *test* benchmark.

Method	Input	Car	Truck	Bus	Trailer	C.V	Ped.	Motor.	Bicycle	T.C	Barrier	NDS	mAP
InfoFocus [40]	Single frame	77.9	31.4	44.8	37.3	10.7	63.4	29.0	6.1	46.5	47.8	39.5	39.5
PointPillars [2]	Single frame	68.4	23.0	28.2	23.4	4.1	59.7	27.4	1.1	30.8	38.9	45.3	30.5
3DSSD [6]	Single frame	81.2	47.2	61.4	30.5	12.6	7.2	36.0	8.6	31.1	47.9	56.4	42.6
PointPainting [41]	Single frame	77.9	35.8	36.2	37.3	15.8	73.3	41.5	24.1	62.4	60.2	58.1	46.4
PointPillars_DSA [42]	Single frame	81.2	43.8	57.2	47.8	11.3	73.3	32.1	7.9	60.6	55.3	59.2	47.0
SSN V2 [43]	Single frame	82.4	41.8	46.1	48.0	17.5	75.6	48.9	24.6	60.1	61.2	61.6	50.6
3DCVF [33]	Single frame	83.0	45.0	48.8	49.6	15.9	74.2	51.2	30.4	62.9	65.9	62.3	52.7
CBGS [44]	Single frame	81.1	48.5	54.9	42.9	10.5	80.1	51.5	22.3	70.9	65.7	63.3	52.8
CVCNet [45]	Single frame	82.7	46.1	45.8	46.7	20.7	81.0	61.3	34.3	69.7	69.9	64.2	55.8
HotSpotNet [46]	Single frame	83.1	50.9	56.4	53.3	23.0	81.3	63.5	36.6	73.0	**71.6**	66.6	59.3
CyliNet [47]	Single frame	85.0	50.2	56.9	52.6	19.1	84.3	58.6	29.8	**79.1**	69.0	66.1	58.5
CenterPoint [19]	Single frame	85.2	53.5	63.6	56.0	20.0	84.6	59.5	30.7	78.4	71.1	67.3	60.3
3DVID [13]	Multi-frame	79.7	33.6	47.1	43.0	18.1	76.5	40.7	7.9	58.8	48.8	-	45.4
TCTR [14]	Multi-frame	83.2	51.5	63.7	33.0	15.6	74.9	54.0	22.6	52.5	53.8	-	50.5
Method of [16]	Multi-frame	86.2	57.2	**67.2**	35.5	14.6	**85.5**	58.1	37.0	71.3	66.2	66.7	59.0
Our TM3DOD (with PP)	Multi-frame	78.8	43.1	52.1	48.1	15.0	71.8	50.2	15.8	59.2	50.3	60.1	48.44
Our TM3DOD (with CP)	Multi-frame	**86.7**	**56.0**	64.6	**58.3**	**27.7**	81.2	**72.7**	**45.2**	77.4	69.3	**69.94**	**63.91**

**Table 2:** The ablation study conducted on the nuScenes *valid* set.

	Motion-Aware Feature Aggregation	Temporal Voxel Encoder	mAP (%)	NDS (%)	Runtime
Single-Frame Baseline			41.18	55.53	31 ms
Our TM3DOD	√ √	√	43.37_↑2.19_ 45.36_↑4.18_	56.85_↑1.32_ 57.93_↑2.40_	44 ms 52 ms

**Table 3:** The ablation study of TCANet conducted on the nuScenes *valid* set.

Modules		Performance	
**Temporal** **Bin Period**	**Temporal-Channel** **Attention**	**mAP (%)**	Δ
-	-	37.6	-
-	√	38.85	+1.25
2	√	39.25	+1.65
5	√	40.27	+2.67
2 and 5	√	40.89	+3.29

## Data Availability

nuScenes dataset at https://www.nuscenes.org/.
